# Informal carers’ experiences in everyday life and the use of digital assistive technology for time management in persons with dementia or mild cognitive impairment

**DOI:** 10.1186/s12877-024-04979-2

**Published:** 2024-04-23

**Authors:** K. Baudin, A. Sundström, H. Lindner

**Affiliations:** 1 Division of Occupational Therapy, Department of Neurobiology, Care Sciences and Society, Karolinska Institutet, Huddinge, Sweden; 2 Department of Health, Medicine and Caring sciences, Division of prevention, rehabilitation and community medicine, Linköping University, Linköping, Sweden; 3 Innovation and Product Realisation, Division of Product Realisation, School of Engineering, Innovation, and Design, Mälardalen University, Eskilstuna, Sweden; 4 School of Health Sciences, Örebro University, Örebro, Sweden

**Keywords:** Dementia, Mild cognitive impairment, Digital assistive technology, Time management, Assistive technology

## Abstract

**Background:**

Digital assistive technology (DAT) may support time management in people with dementia or mild cognitive impairment (MCI), but research on DAT for time management is limited. We aimed to explore how everyday could be supported by DAT for time management in persons with dementia or MCI from informal carers’ perspectives. This study focused on a DAT device for time management called MEMOplanner (MMP).

**Method:**

Using a mixed-methods design, we utilized the Time-Proxy© questionnaire and a study-specific interview guide to investigate the perspectives of informal carers (*n* = 8) regarding the use of MMP by individuals with dementia or MCI.

**Result:**

The MMP was helpful in keeping track of time and activity. It helped to maintain an active lifestyle and facilitated communication. However, the MMP did not reduce the need for assistance from the informal carers, and it took time to learn the different functions of the device. Further research into employing a more extensive array of DAT for time management or other areas to assist individuals with dementia will yield valuable insights into enhancing and sustaining a higher quality of life despite cognitive decline.

## Background

Older adults with dementia or mild cognitive impairments (MCI) may experience symptoms such as forgetfulness, communication difficulties, confusion, changes in mood or behavior, and challenges in carrying out familiar tasks [1]. These symptoms often result in reduced independence and the ability to self-care.

From a neurodegenerative perspective, the irreversible loss of neurons in individuals with dementia continues to drive a gradual decline in a wide range of cognitive functions, such as attention deficits, slow processing speeds, deficits in short- and long-term memory and executive dysfunction that impaired our ability for time management [2–4]. This gradual cognitive decline presents everyday challenges for individuals with early dementia in their immediate environment. These challenges include disturbance, physical inactivity, impaired decision-making, poor financial management, and impaired memory for planned events [5–8].

The inability to keep track of time, a change in the quality of life, and the need for assistance are common in persons with early dementia and MCI [9, 10]. Keeping track of time is managed by our brain function “time perception”, which is the capacity to perceive, judge, and represent time intervals [11]. Disturbances in time perception in persons with dementia or MCI have severe consequences in daily life [12].

From an aging-in-place perspective, people with dementia or MCI prefer to live at home for as long as possible despite experiencing gradual cognitive decline [13]. An estimated 60%, 62%, and 64% of persons with dementia live at home in Sweden, in Europe and the United States [14–16] The question becomes: How can we cope with the increasing number of individuals with early dementia who prefer to remain in their own homes? Currently, pharmacological treatments available for dementia offer inconsistent results and may have undesirable side effects [17], making the search for evidence on non-pharmacological interventions to support individuals with dementia or MCI of significant interest.

Different non-pharmacological interventions include reminiscence, validation, and cognitive stimulation therapies [18]. One promising non-pharmacological intervention for supporting cognitive decline is digital assistive technology (DAT) [19]. A common category of DAT is digital memory devices, which are used to compensate for gradual memory impairment. Several reviews have reported the usefulness of DAT in supporting cognitive decline [13, 20–22], such as self-management in everyday activities, social participation, and the prevention of loneliness.

Although the usefulness of DAT is frequently reported in intervention studies, there have been relatively few mixed-method studies that investigate the usefulness of DAT for time management in persons with dementia or MCI from an informal carer perspective. An informal carer is an individual person who offers unpaid assistance to a friend or family member needing support [23]. A mixed-methods research approach is beneficial because it combines the strengths of qualitative and quantitative research methods. This approach allows researchers to gain a more comprehensive and nuanced understanding of a research problem. Previous mixed-method studies that reported the usefulness of DAT mainly focused on well-being [24], counselling [25], social participation [26], and activity monitoring [27]. However, there is a lack of mixed-method studies focusing on time management. Being able to manage time facilitates continuing living at home. The study’s aim was to explore how DAT could support everyday time management in persons with dementia or MCI from informal carers’ perspectives. Two research questions were asked: (i) How was the experience of living with cognitive decline? And (ii) how did DAT support time management in the daily life of persons with dementia or MCI?”

## Methods

### Study design

A mixed-methods study design with a questionnaire and semi-structured interview was used to answer the research questions. Specifically, a Convergent parallel mixed method was used to collect the quantitative and qualitative data on one occasion and to analyze them separately [28]. This design enables researchers to comprehensively understand a research problem by leveraging the strengths of qualitative and quantitative methods. The qualitative and quantitative data are collected concurrently but analyzed separately, providing a rich and deep exploration of the phenomenon [29].

### MEMOplanner (MMP)

**Fig. 1:**
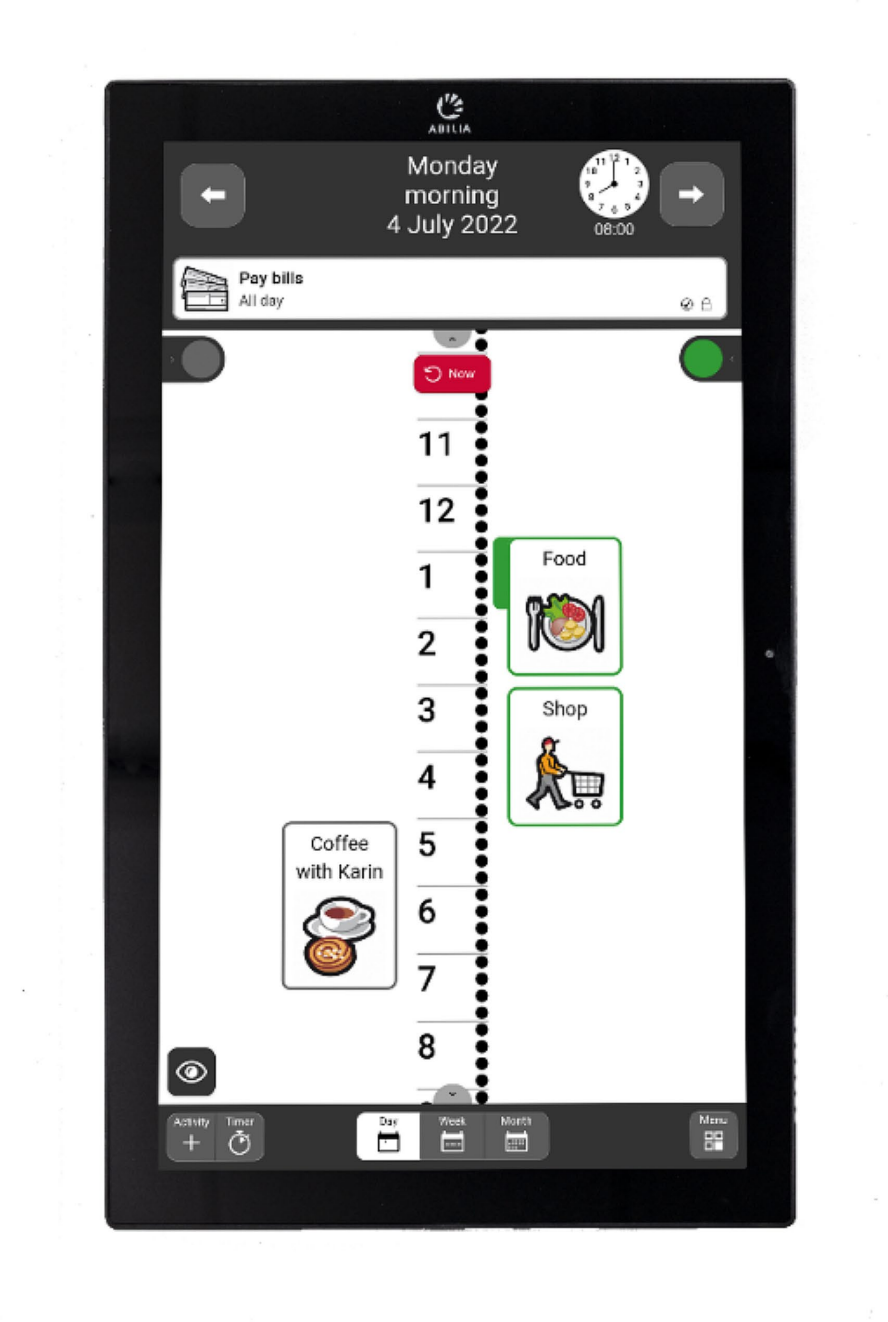
**.** A graphical interface of a MEMOplanner

The DAT we investigated in this study is MEMOplanner (MMP) [30] (Fig. 1). It is a digital calendar and is one of the most commonly prescribed DATs for persons with dementia during the last five years in Sweden (unpublished data). The MMP displays a calendar with two timelines, one for daytime hours (light area) and one for night-time hours (dark area). The light area reminds the user that it is daytime and to stay awake, whereas the dark area reminds the user that it is time for bed and sleep. It provides the current time and an overview of the day, week, and month. The voice reminder function of the MMP notifies its user when a task or event is about to begin or end. The voice reminder functions as a reminder of whether the user has completed a specific task or for the timing of a specific event. It has a built-in speech function, and animated images of tasks/events can be added along the timeline.

### Participants

The potential study participants were informal carers of the MMP users. They were recruited using stratified purposive sampling [31] via the assistive technology center in Stockholm, Sweden. The assistive technology centers in Sweden are part of the public healthcare services that provide assistive technologies for persons with various functional impairments. The inclusion criteria for participants in this study were as follows: an informal carer who (i) regularly visited or lived together with a person diagnosed with dementia or MCI, and (ii) the person with dementia or MCI was currently using an MMP to support cognitive decline, specifically, a regular MMP user for at least six months. The recruitment process was conducted from fall 2020 to winter 2021, resulting in eight informal carers as study participants.

### Ethical considerations

The Regional Ethical Review Board in Sweden approved the study on 23 June 2020 (approval number 2020 − 02007). Approval was given for collecting data from both persons with dementia and their carers. During the interview period (fall 2020 to winter 2021), because of COVID-19, the Swedish ethical authority recommended us to ask for consent from persons with dementia to interview their informal carers. Contact with people with dementia was not recommended due to an increased risk of contracting the virus. Therefore, we decided to recruit informal carers with the consent of the persons with dementia. Following the ethical procedure, voluntary participation was emphasized in the information. Both verbal and written information were provided before they gave their consent. The collected data was kept confidential. All data were analyzed collectively without individual identification.

### Questionnaire

A validated and reliable questionnaire, Time-Proxy© was used to collect the informal carers’ perspectives regarding time management of the MMP users [32]. It consists of 12 items that focus on three significant areas: bedtime routine, keeping track of time for daily events, and keeping track of non-daily events and future task planning. All items are rated on a 5-point Likert scale: *never, sometimes, often, and always*, with an extra option: *do not know.*

### Interview guide

A study-specific interview guide was designed to interview informal caregivers, and the guide has two parts. Part 1 consists of questions regarding the demographic characteristics of the informal carer, for example, if the informal carer is the spouse, child, or sibling of an MMP user and if the carer is accustomed to everyday digital technology. Part 2 includes nine questions that focus on three main areas: (i) the estimated time spent supporting the user to use the MMP and the estimated daily time the user used the MMP; (ii) perceived the need for assistance provided by domestic service after using the MMP; (iii) perceived everyday life, relationship with the user, and quality of life after using the MMP.

### Data collection procedure

Via the assistive technology service in a big city in central Sweden, the first author (KB) obtained the contact details of the persons who had been prescribed MMP during 2019–2020. The first author contacted the MMP users to provide information about the study and to get their consent to collect data from the informal carers. In the next step, the first author contacted the informal caregivers to provide information about the study and decide the dates for telephone interviews. The informal carers were informed verbally of the study’s aim and their rights to withdraw at any time, and they were given their written consent before the interviews. Eight interviews were conducted from October 2020 to May 2021, taking around 25 to 45 min.

### Data analysis

**Fig. 2:**
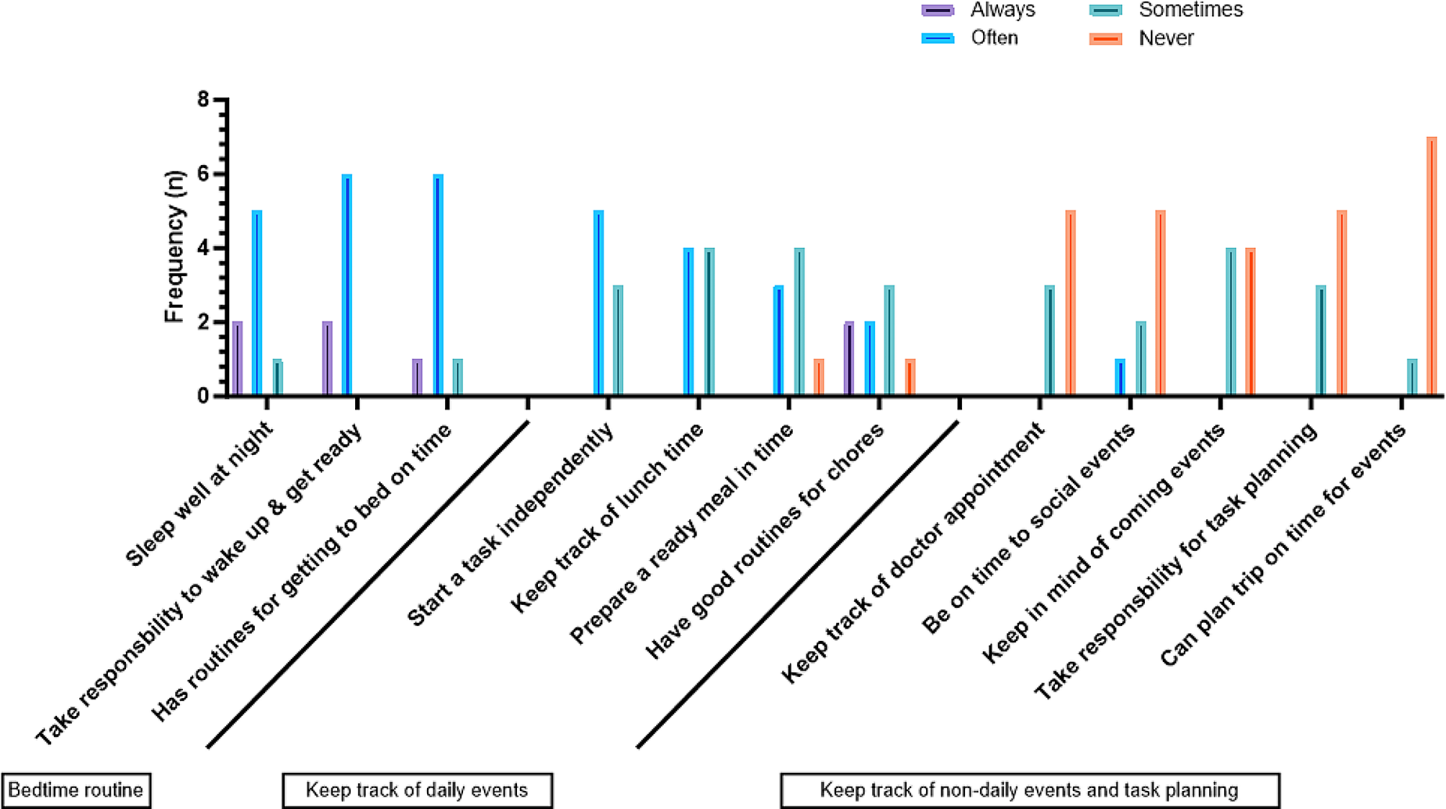
Informal carers’ responses on daily time management of the MEMOplanner users (*n* = 8)

The data from Time-Proxy© was analyzed using Excel, and the GraphPad software was used to present the result using a bar chart (Fig. 2). The interviews were analyzed by following the steps using inductive thematic analysis [33]. The first author (KB) conducted the interviews and gathered the data for questionnaires. The transcribed interviews were then read several times to achieve an overall understanding of the data, and the analysis process then continued with identifying and underlining the codes, followed by condensing and abstracting the codes into themes (by KB). Whereby similarities and differences that guided the organization of themes were discussed with the other two authors until a consensus was reached. Finally, two themes and six subthemes related to the study’s aim were identified.

## Results

### Demographics of the MMP users and the informal carers

Among the eight MMP users, six out of eight have dementia, and two had MCI (Table 1). Their average age was 64.6 years old, and five were female. Six of eight MMP users lived with someone, and five out of eight were accustomed to everyday digital technology, such as mobile phones.

**Table 1:** Demographic characteristics of participants with dementia or mild cognitive impairments

Characteristics		*N* = 8
Age, mean (SD)		64.6 (9.1)
Sex	Male Female	3 5
Diagnosis	Early stage of dementia Mild cognitive impairment	6 2
Living condition	Together with someone Alone	6 2
Domestic service*	Yes No	4 4
Education level /Years of education	University Gymnasium Unknown	2 5 1
Accustomed to everyday digital technology	Yes No	5 3
MMP use time	Never Once per week Several times per week Once or twice per day Several times per day	0 0 4 4 0

*Community staff provide different domestic services for the users

The participants’ average age was 60.9 years old, and four were female. Six of them were still working. Six out of eight informal carers were the spouses of the MMP users (Table 2).

**Table 2:** Demographic characteristics of informal carers

Characteristics		*N* = 8
Age, mean (SD)		60.9 (8.7)
Sex	Male Female	4 4
Relationship with the MEMO Planner user	Live together/married Child Nephew	6 1 1
Education level/years of education	University Gymnasium 14–17 years	4 1 3
Occupation status	Full time or part time Retired	6 2
Accustomed to digital technology	Yes No	8 0

### Time management

All informal carers (*n* = 8) filled in the Time-Proxy© regarding how the MMP users have used the MMP for time management. All MMP users have been using the MMP for at least six months. The results are presented in Fig. 2. Concerning bedtime routine (Fig. 2, the left 3 columns), seven MMP users always or often go to bed on time, bedtime, and wake up on time.

Regarding the questions on keeping track of daily events (Fig. 2, the middle four columns), five MMP users often or two users can sometimes start a task independently. Around four of them often, and four sometimes, can keep track of lunchtime. Four of them often, or three of them sometimes, can prepare meals in time. Seven MMP users have good routines for chores.

Regarding the questions on keeping track of non-daily events, such as doctor’s appointments or social events, and planning for future events (Fig. 2, the right 5 columns), four MMP users can never keep track of doctor’s appointments, never be on time for social events, and never keep in mind coming events. In terms of task planning, six MMP users were never able to take responsibility for future task planning and plan trips on time for events.

### Semi-structured interviews

Two themes and six subthemes emerged from the interviews (Table 3). One theme describes everyday life challenges due to cognitive decline as perceived by informal carers, which is divided into subthemes; *keeping track of time and activity, quality of life, and the need for assistance due to cognitive decline.*

**Table 3:** An overview of the themes and subthemes

First theme	Second theme
Everyday life challenges due to cognitive decline	The use of DAT for time management
Subthemes	Subthemes
• Keeping track of time and activity	• A visual reminder with an alarm to enable an active lifestyle
• Quality of life	• A tool to create new bonds for communication
• The need of assistance	• The process of adapting to technology

The other theme describes the use of DAT for time management, which is divided into three subthemes; *a visual reminder for maintaining an active lifestyle, new bond for communication and the process of adapting to DAT*.

### Everyday life challenges due to cognitive decline

This first theme emerged from the informal carers’ perceptions of challenges due to cognitive decline, and it is supported by three subthemes: *everyday life, quality of life, and assistance due to cognitive decline.*

### Keeping track of time and activity

This subtheme emerged from the data, as the majority expressed that the cognitive decline of the users imposed challenges in everyday life.

*“We live and do stuff together. It is terribly sad what such an illness can do to it. She was so active.”*

Another challenge was the decline in the ability to keep track of time. Often, the users do not engage in much activity during the days when they are alone at home.

*We live together, and I work, and she mixes things. If you take “keep tracking of hours” as an example, it is already a lot for her. Before the illness, she managed time without a problem, and now she manages to keep track of two to three hours per day, but it varies depending on her status*.

In addition, some expressed sadness that the person is affected by a progressive illness.

*It generally works in everyday life, but it’s hard, of course. Sometimes you think about how it will turn out in the future, but we try to take one thing at a time. We will see how everything develops as time goes on*.

### Quality of life

This subtheme that emerged from the interviews was the informal carers’ perception of quality of life due to cognitive decline. The illness is something that affects not only the individual who has been diagnosed but also their immediate surroundings. Some of the users in the study are young (52 years old), which means that their partners are of working age, and they tried to manage the user’s symptoms and necessary appointments while at the same time managing other essential things in life such as work, children, exercise, and social life with friends and acquaintances.

*The illness controls you; yes, I rush home to her after work because it does not feel good otherwise, and so on.*

One younger informal carer (under age 60) expressed that they are still processing the shock about the illness and how it affects their partners, whereas the older informal carers are more accepting that illnesses come with age and that they take the day as it is.

*It is tough. Really tough. I am worried about the future. He will have to live somewhere else. I must work; I am young, and he is young, too. So far, we can manage it. The children also help and come by if there is anything, but yes, the quality of life has become very different.*

Some informal caregivers shared about how their daily lives were affected due to their partners’ cognitive declines and how they must adapt in different ways in their everyday lives. One informal carer expressed the following about the user’s quality of life and situation:

*It is okay to adapt. We noticed early on that something was not right long before he received a diagnosis, but we thought he was just stressed and careless. Luckily, we have our children and friends. He must motivate himself to cope, but he mostly sits at home. He does not want anything and just says he is so tired.*

### The need for assistance

This subtheme derives from the fact that due to cognitive decline among the MMP users, all informal carers provided physical help and assistance in daily living to the users. The amount of support and assistance varies depending on whether the user lives with the participant, such as a partner or spouse, or whether the participant is a relative who frequently visits the user and helps with whatever is needed.

*I am the one who lives near him, so I usually drop by every weekend; my mom talks to him and checks how he is doing, but I go there and do things. I check on him, cook, shovel snow, fix things, and help him with various things. Maybe two to three hours a week in total. I usually call him now, since he got the illness, a few times a week. So, it is actually a few hours of help.*

Some informal carers tried to get their spouses to continue with various activities they knew the users had done before, such as something in the kitchen, carpentry, or fixing the garden. One participant said:

*I am trying to get him to continue doing things that he has always been good at before so that he continues to do things like that.*

### The use of DAT for time management

The second theme emerged from the informal carers’ expression that time management had significantly improved since the users started using the MMP. The participants pinpointed that MMP is a clear and structured calendar, and they have used the MMP to create a daily schedule so that the users can continue to be as independent as possible in everyday life. The MMP has been used to provide an overview of upcoming activities in the next few days, such as transport services, appointments at the dentist, etc. Some informal carers also expressed that, thanks to the MMP, the time schedules in everyday life are more precise, and everyone in the immediate surrounding area feels better because of this, including the MMP user. The three subthemes that emerged were *a visual reminder with an alarm to enable an active lifestyle, a tool to create new bonds for communication, and accustoming to everyday technology.*

### A visual reminder to maintain an active lifestyle

The majority said that the MMP provides a visual reminder for the users when, for example, they must go somewhere by themselves in the morning. The users used the MMP as reminders of what was going to happen that day and in the next few days.

*We use it to get a good overview of days, times, and so on, it can provide reminders… I am still working, so she must know if I am off duty, at work, or away on something.*

They expressed that the user frequently uses MMP, the informal carers themselves, and the domestic service staff. One of the younger users received reminder alarms via the MMP and could thus prepare himself and travel with the transport service to his daily business or meeting.

One participant informed us that if his spouse was not at home to set an alarm reminder for him, he would often feel tired and inclined to sit on the couch in front of the TV. The participant highlighted that receiving the alarm reminder facilitated a more active lifestyle, resulting in an improved daily routine, better sleep at night, and a greater sense of well-being throughout the day.

In some cases, the MMP’s reminder function helped to prevent users from asking the informal carers repeatedly about time and what was going to happen, which meant that the participants were encouraged to look at the MMP and tried to incorporate it into the individual’s daily routines and habits. However, some of the informal carers doubted whether the users would use the MMP when they were alone because they knew that the person often forgot about time and sat in front of the television.

*She keeps asking what we should do now, and we try to get her to use the calendar to keep track of different events, but I do not know how much she uses it herself when she is alone during the day.*

Regarding the hours of assistance, most informal carers said that the MMP did not minimize the support they provided or the number of hours for domestic services. However, the visual reminder was a good help in structuring the day and helping the user be more independent.

### Facilitator for communication

The second subtheme showed that by talking about time and activities in the coming days, the informal carers felt that the users were able to maintain their participation in everyday life. One participant talked about how she talked to her mother about how MMP visualizes different activities on different days. A participant described the following:

*We talk in a different way today, maybe partly because of the calendar, about what the days will look like, what will happen, and things you look forward to. Yes, a bit like when the children were small; it is positive, I think, for the whole family.”*

Some informal carers expressed that the MMP is part of their communication with the users. They used the MMP to communicate with the users about the activities during the day and talk about the activities in the coming days.

*We talk more about the calendar. I encourage my mother to look at the calendar to remind her of the various activities that are to take place, for example, the hairdresser’s appointment on Friday or something similar. We will do that*.

*Yes! After all, he is alone during the day or goes to a day centre a few times a week to have something to do. We use the MMP to talk about what he should do every day when he should go with the transport service, what is going on at the weekend, and so on.*

### The process of adapting to DAT

The last subtheme that emerged is the process of adapting to DAT. All informal carers expressed that they use everyday technology regularly, partly due to daily computer use and partly due to daily use of smartphones. The informal carers who are currently working expressed that they use the computer for work tasks and use their smartphones to take photos, listen to music, and use internet banking services. One participant expressed the following about his daily use of technology:

*“Yes, I consider myself relatively tech-savvy; I use the computer every day in different ways, at work, checking things up. Yes, you live with your laptop under your arm these days and are dependent on it! Using e-mail, banking services, music, listening to books, etc. on a smartphone*.

Even though most informal carers are accustomed to everyday technology, they have not invested time to learn all the functions of the MMP and its potential. To informal carers, it is a “calendar” that the user has hung up in their kitchen, and it looks like a calendar.

## Discussion

The study aimed to explore how DAT could support everyday activities for time management in persons with dementia or MCI from informal carers’ perspectives. The MMP is a useful time management device that supports daily routine, keeps track of daily events, maintains an active lifestyle, and facilitates communication. However, the MMP did not reduce the need for assistance from the informal carers, and it took time for the informal carers to learn the different functions of the device.

The results showed that MMP is an excellent visual reminder, but it does not reduce the support from the informal carers. The extent of carers’ burden has been extensively investigated in the literature, and our result adds one more piece of evidence to this phenomenon [34]. One possible reason could be that the carers were unsure if the MMP users would take the initiative to look at the MMP.

The persons with dementia or MCI receive the MMP from the assistive technology centers, and the therapist also provides 1- or 2-hour training with written instructions to the person and the carers. The therapist usually provides basic function training first and does follow-up if the user wants other functions. The results showed that the informal carers did not have the time to learn other functions.

Despite the small sample, the findings from the Time-Proxy© and the interviews suggest that the MMP has the potential to encourage a good day and bedtime routine. A good day and bedtime routine promotes sleep quality, which has a crucial role in preserving memory, and sleep disturbance is common in dementia and MCI [35]. In light of a lack of effective medication for sleep in dementia [36], this finding shows that the DAT is a beneficial non-pharmacological intervention. Moreover, sleep medication often has considerable side effects, such as increased hospitalization and falls in persons with dementia [36]. In contrast, DAT does not generally induce any side effects or increase the risk of medication compliance, which is a common risk in older adults [37].

The functions of MMP encourage users to continue an active lifestyle. The MMP can be seen as a tool to enable people with dementia to stay mobile and maintain their functional independence. Furthermore, sleep has an essential health promotion function [38] because sleep deprivation significantly reduces cognitive and motor performance [39]. By encouraging the continuation of physical activity, the MMP indirectly improves the user’s bedtime routine because physical activity during the day sets the stage for a good night’s sleep. Furthermore, as retirement ages rise across high-income countries, society’s interest in dementia and working life is increasing, suggesting that the DAT may play a crucial future role at work for employees with early dementia.

Regarding daily events, the quantitative and qualitative findings show that the MMP supports daily activities such as preparing meals on time and doing chores. The MMP clearly displays everyday schedules on the display, such as when to start preparing meals and when to start doing chores such as washing or cleaning. Being able to continue performing activities of daily living (ADL) in their own homes is a strong indicator of dependence for people with dementia who prefer to live in their own homes. From an ageing-in-place perspective, the functions of MMP support the wish of people with dementia to stay longer in their own homes.

In terms of non-daily events and further planning, however, the finding from Time-Proxy© suggests that the MMP may work effectively as a tool for reminders. However, the MMP users may not always act upon the reminder or alarm. Future events or task planning are managed by the brain function “mental time travel” [11]. Mental time travel refers to the capacity to mentally navigate through subjective time, including episodic memory and episodic foresight or future thinking. This vital brain function enables us not only to go back in time but also to foresee, plan, and shape virtually any specific future event However, the therapists who prescribed the MMP to the user could train the informal carers in how to break down the steps of non-daily events or further planning and register the steps in the MMP before the actual date of a future event. For example, for a birthday party planning, the carer can enter steps in the MMP in advance, such as buying party decorations or writing invitations, two weeks before the actual party date.

All informal carers in this study expressed that there had been a significant change in time management since the users started using the MMP. This implies that the MMP helps the user become independent and, thereby, less dependent on their informal carers to remind them about upcoming activities and tasks to be done. Previous research suggests that it is essential to provide people with dementia or MCI with DAT as early as possible for the best possible improvement or support in time management [12, 40]. Furthermore, even though most of the informal carers were accustomed to everyday digital technology, they had not invested time to learn all the functions of the MMP. Early prescription of DAT with structured training and follow-up will enable the users and informal carers to explore the full potential of DAT.

One surprising finding is that the MMP facilitates communication between the informal carer and the user. The people with dementia felt disconnected because of the illness, but because of the MMP, they became connected to the outside world again. According to the findings, the MMP provides discussion topics between the informal carers and the users (e.g., the MMP itself, upcoming activities, and schedule), which facilitate meaningful daily conversation. In this way, the MMP helps the informal carers stay informed and supports exchanging daily information about the users. It may be beneficial to give the MMP a name, just like a family pet, so that the MMP becomes a digital family member.

### Methodological considerations

One main study limitation is the small sample in this exploratory study, like other studies that evaluated technology in older people or patients [41–43]. The researchers vary in terms of professional background (occupational therapist and economist) and experience of the target group, as well as qualitative research, which strengthens trustworthiness [44]. Moreover, the convergent parallel mixed-methods design offers the benefits of methodological diversity, triangulation, and comprehensive insight, making it a valuable approach for researchers seeking a more thorough and nuanced understanding of the research problem [29].

The interviews provided an opportunity to explain and dive deeper into the findings of the questionnaire that explored the impact of DAT use on time management. One study strength was the recruitment of informal carers via the staff at the assistive technology center, which enabled self-chosen recruitment for the study. Five out of eight MMP users are accustomed to technology; hence, the results might limit the transferability of our findings to MMP users who are not accustomed to technology. Furthermore, we chose to evaluate one DAT in this study to facilitate interpreting the results based on the functions of the MMP. This choice limits our findings to other DAT uses in people with dementia. Interviews about past events carry a risk due to the challenge people face in accurately recalling details. Individuals may inadvertently provide inaccurate information [45]. This may be frustrating for the informal carers who gave interviews.

## Conclusion

The results showed that MMP was an excellent visual reminder, but it did not reduce the support from the informal carers. The extent of carers’ burden has been extensively investigated in the literature, and our result adds one more piece of evidence to this phenomenon [34]. One possible reason could be that the carers were unsure if the MMP users would take the initiative to look at the MMP. Despite cognitive decline, the use of DAT supported people with dementia or MCI to have a structured daily routine. However, DAT did not minimize the support from informal carers and domestic services. Further research into utilizing a broader range of Digital Assistive Technologies (DAT) for tasks such as time management and other areas could provide valuable insights into improving and maintaining a better quality of life for individuals with dementia, despite cognitive decline.

## Data Availability

The datasets used and/or analyzed during the current study are available from the corresponding author upon reasonable request.
