# Correction: Informal carers’ experiences in everyday life and the use of digital assistive technology for time management in persons with dementia or mild cognitive impairment

**DOI:** 10.1186/s12877-024-05077-z

**Published:** 2024-06-21

**Authors:** K. Baudin, A. Sundström, H. Lindner

**Affiliations:** 1 Division of Occupational Therapy, Department of Neurobiology, Care Sciences and Society, Karolinska Institutet, Huddinge, Sweden; 2 Department of Health, Medicine and Caring Sciences, Division of Prevention, Rehabilitation and Community Medicine, Linköping University, Linköping, Sweden; 3 Innovation and Product Realisation, Division of Product Realisation, School of Engineering, Innovation, and Design, Mälardalen University, Eskilstuna, Sweden; 4 School of Health Sciences, Örebro University, Örebro, Sweden

BMC Geriatrics (2024) 24:365

10.1186/s12877-024-04979-2

After publication of this article, the authors reported that in Table 2, a table note was erroneously added, and should be removed.

The original article has been corrected.

